# Differences in Gene Expression between Mouse and Human for Dynamically Regulated Genes in Early Embryo

**DOI:** 10.1371/journal.pone.0102949

**Published:** 2014-08-04

**Authors:** Elo Madissoon, Virpi Töhönen, Liselotte Vesterlund, Shintaro Katayama, Per Unneberg, Jose Inzunza, Outi Hovatta, Juha Kere

**Affiliations:** 1 Department of Biosciences and Nutrition; Center for Biosciences, Karolinska Institutet, Huddinge, Sweden; 2 Department of Obstetrics and Gynecology, Karolinska University Hospital, Stockholm, Sweden; 3 Department of Medical Epidemiology and Biostatistics, Science for Life Laboratory, Karolinska Institute, Stockholm, Sweden; 4 Karolinska Institute, Department of Clinical Science, Intervention and Technology, Karolinska University Hospital K57, Stockholm; 5 Molecular Neurology Program, Research Programs Unit, University of Helsinki, and Folkhälsan Institute of Genetics, Helsinki, Finland; Universitat Pompeu Fabra, Spain

## Abstract

Infertility is a worldwide concern that can be treated with *in vitro* fertilization (IVF). Improvements in IVF and infertility treatment depend largely on better understanding of the molecular mechanisms for human preimplantation development. Several large-scale studies have been conducted to identify gene expression patterns for the first five days of human development, and many functional studies utilize mouse as a model system. We have identified genes of possible importance for this time period by analyzing human microarray data and available data from online databases. We selected 70 candidate genes for human preimplantation development and investigated their expression in the early mouse development from oocyte to the 8-cell stage. Maternally loaded genes expectedly decreased in expression during development both in human and mouse. We discovered that 25 significantly upregulated genes after fertilization in human included 13 genes whose orthologs in mouse behaved differently and mimicked the expression profile of maternally expressed genes. Our findings highlight many significant differences in gene expression patterns during mouse and human preimplantation development. We also describe four cancer-testis antigen families that are also highly expressed in human embryos: PRAME, SSX, GAGE and MAGEA.

## Introduction

Infertility is a significant medical problem affecting tens of millions of couples worldwide [1]. In vitro fertilization (IVF) is commonly used to treat infertility, but improvements are still needed as indicated by the low live-birth rate of 32% [2]. The IVF treatment includes culturing of the human embryo up to the whole preimplantation period, covering many crucial steps in the early embryo development: the fusion of the oocyte and sperm pronuclei at 1-cell stage, maternal transcript degradation, activation of the zygotic genes at 4- and 8-cell stages and lineage decisions in the blastocyst stage. It is necessary to understand better the molecular mechanisms of preimplantation development in order to improve infertility treatment.

Global gene expression studies in human have identified thousands of genes expressed in human oocytes and preimplantation embryos [3]–[11]. Maternally loaded genes are downregulated before the blastocyst stage and include genes essential for oocyte maturation and embryo development, such as *HSF1* [12] and *NLRP*5 [13]. However, up to 45% of genes detected in oocytes have unknown functions [8], highlighting maternally loaded genes as important candidates for functional research. The start of gene transcription in embryo, called the zygotic genome activation (ZGA), takes place in the 4- and 8-cell stages in human [8], [11], [14] and in the 1- and 2-cell stage in the mouse [15], [16]. ZGA includes the transcription of known genes important for pluripotency, embryo development and lineage specification, such as *NANOG* [17], [18].

Mouse is a common model organism used for understanding the function of genes in preimplantation development [19]. Although both similarities and differences between mouse and human global gene expression patterns have been described using genome-wide experimental approaches [10], [11], [20], differences or similarities of genes for human and mouse early development still need verification.

We aimed to identify genes relevant for human preimplantation development and study the expression of these genes in the mouse. We used two independently published microarray expression datasets for human preimplantation development [8], [11] and online databases to define the genes of interest. Expression clusters of upregulated genes such as *NANOG*, and downregulated genes such as *NLRP5* were identified. In addition, we studied genes that are activated in ZGA and thus upregulated in mouse by 2-cell stage [10], [11]. We show that 29 out of 30 downregulated genes share an expression profile between human and mouse, whereas the expression profile differs for 16 upregulated genes out of 25. These results indicate that there are species differences between human and mouse early gene expression that might affect the interpretation of the results obtained in mouse as a model organism.

## Materials and Methods

### Microarray analysis

Raw data for human preimplantation embryos on Affymetrix GeneChip HGU133 Plus 2.0 were obtained from ArrayExpress, accession numbers E-MEXP-2359 [8] and E-GEOD-18290 [11]. Arrays were analyzed as previously described [8]. Briefly, the invariant set normalization method was used and expression values were extracted from PM-values using the Li-Wong method [21]. Arrays were normalized independently, rescaled to the same median intensity and the Li-Wong method was applied to all the normalized arrays together to get summary expression measurements. Data from the following stages were used in this study: MII, 4-cell, 8-cell and blastocyst from Zhang et al. (2009) and 1-cell, 4-cell, 8-cell and blastocyst from Xie et al. (2010). The analysis of differential expression between the consecutive developmental stages was performed using a Bayesian approach [21], [22] as implemented in the Limma package (www.bioconductor.org). Differential expression p-values reported were corrected for multiple testing using the FDR method and q-values less or equal to 0.05 were considered significant. No cut-off value was set for fold-change. In order to display results comparatively with qPCR data, the expression values called log2(comparative expression) were obtained as follows: log2(comparative expression)  = log2[gene]-log2[average controls], where [gene] is the value of a certain probe for the gene and [average controls] is the mean value of probes for endogeneous controls *Hprt1* (202854_at) and *Psmb6* (208827_at). Gene names with corresponding probesets are listed in Table S1.

### Embryo collection

FVB/N mice were kept under 12 h light/dark cycle and had free access to food and water. 4–7 weeks old females were injected with 5 IU i.p. Pregnant Mare's Serum (Folligon, Intervet) followed 44 h later by 5 IU i.p. injection of human chorionic gonadotropin (hCG) (Chorulon, Intervet). Females were mated with male FVB/N strain studs. The females were sacrificed 19–21 h later by cervical dislocation and the oviducts were collected in M-2 medium (Millipore). Cumulus cells were removed by 0.3 mg/ml hyaluronidase treatment (Sigma-Aldrich). Oocytes or 1-cell embryos were collected 21–23 h after hCG. Embryos were cultured in KSOM medium (Millipore) under ovoil-100 (Vitrolife) until 2-cell (45–47 h after hCG) and 8-cell (71–73 h after hCG) stages.

### Gene expression analysis

qPCR was performed using Custom TaqMan Low Density Array Cards. RNA from mouse unfertilized oocytes (MII), 1-cell embryos (1-cell), 2-cell embryos (2-cell) and 8-cell embryos (8-cell) was extracted using Arcturus PicoPure RNA isolation kit (Applied Biosystems) according to manufacturer's instructions using optional DNase treatment with RNase-Free Dnase (p/n 79254, Qiagen). RNA quality and concentration were measured by Agilent Bioanalyzer using Agilent RNA 6000Pico Kit. One oocyte or embryo yielded 128 pg of total RNA on average. Samples of 12 or 5 ng of RNA for each sample were converted to cDNA using High Capacity cDNA Reverse Transcription Kit (Invitrogen) according to the manufacturer's instructions. An additional 5 ng of RNA for replicas in each stage was treated similarly, except that oligo(dT)_20_ primer (Invitrogen, 55063) was used instead of random hexamers provided with the cDNA synthesis kit. cDNA was mixed with TaqMan Universal PCR master mix (p/n 4304437, Applied Biosystems (ABI) Foster City, CA, USA) and RNase-free water. Two loading ports were used per sample and 100 ul was loaded into each of the 8 ports. The array was sealed and centrifuged for 2 min at 1200 r.p.m. and loaded on qPCR machine 7900HT (ABI, Singapore) with ABI software SDS v2.4. Standard TLDA array cycling was used. Additional 5 ng samples with random hexamers cDNA synthesis were pre-amplified. Array specific custom TaqMan pre-amp pool (Invitrogen) was used for pre-amplification of the cDNA prior loading to cards according to manufacturer's instructions. Three biological replicas of all stages were collected for each protocol, except for the 12 ng protocol, where two replicas for both MII and 1-cell samples were used instead of three.

### TaqMan Array Cards analysis

Ct values were analyzed using RQ Manager version 1.2.2. (Applied Biosystems). Automatic threshold was set and subsequently adjusted by using manual threshold where needed. One assay (*Rfpl4b*) did not pass our quality criteria and was thus excluded from further analysis. ΔCt values were obtained using DataAssist Software version 3.0 (Applied Biosystems). The endogenous controls *Hprt1* and *Psmb6* were used for normalization. Nanog and Nlrp5 were used as positive controls for “Up” and “Down” clusters, respectively. The Ct value 40.0 was included in the calculations for not detected transcripts. The lowest calculated −ΔCt value in the samples in the same protocol was set for all the not detected transcripts in this protocol. −ΔCt values for undetected samples were not included in the calculation of average values for plotting, unless all replicas were undetected. Changes in the expression between MII vs 1-cell, 1-cell vs 2-cell, and 2-cell vs 8-cell were calculated using student's t-test when at least two replicas were detected in both stages. p-values equal to or less than 0.05 were considered significant (Table S1). heatmap.2 function from gplots package in R was used for drawing heatmaps.

### Expression analysis from public sequencing dataset

Normalized RPKM (reads per kilobase per million) values for human and mouse preimplantation stages were obtained from the Gene Expression Omnibus database (GSE44183_human_expression_mat.txt.gz, GSE44183_mouse_expression_mat.txt.gz) [10]. p-values and ratios were calculated for pairwise comparisons between oocytes and 4-cell blastomeres and oocytes and 8-cell blastomeres in human after addition of 0.1 to every value. p-values equal to less than 0.05 were considered significant. Genes that were upregulated more than 5 times by 4-cell or 8-cell embryo, were used for ortholog search in mouse. Mouse orthologs were obtained from the Biomart database by using human gene names as query. The upregulated human genes and their mouse orthologs along with expression values are shown in Table S2. Average values for each stage in human and mouse were calculated between the cells or embryos from the same biological stages. Number 1 was added to each value before logarithmic transformation of the data for plotting, resulting in values ln(RPKM+1).

### Ethics Statement

The use of experimental animals and the research protocol in this study were approved by the appropriate Animal Care Board (Jordbruksverket), ethical permits S137-10 and S167-11. The animals were treated in accordance with Swedish law and the regulations of Karolinska Institutet.

## Results

### Identification of three expression clusters: “Up”, “Up-down” and “Down”

**Figure 1:**
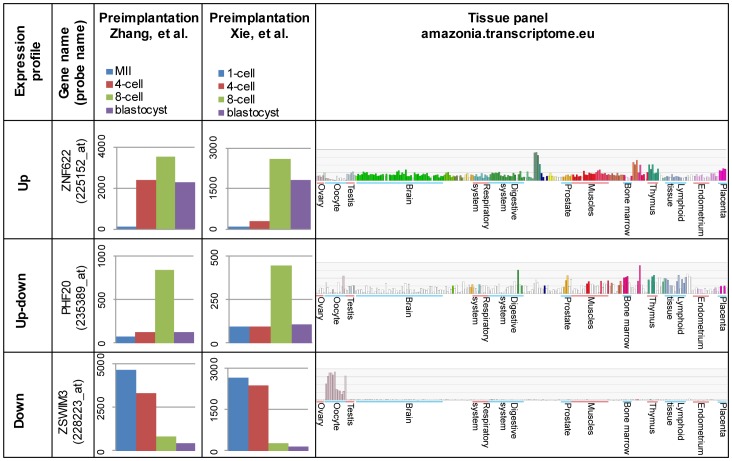
Examples of genes from the three different expression profile clusters. Three different expression profiles are shown for three genes: “Up” (*ZNF622*), “Up-down” (*PHF20*) and “Down” (*ZSWIM3*). Average gene expression from normalized arrays are shown for two independent preimplantation microarray sets: Zhang et al. (2009) and Xie et al. (2010). Expression for various human tissues from Amazonia database show typical examples of selected genes. The larger groups of tissues are labeled, more information about the samples can be found from Amazonia database http://amazonia.transcriptome.eu/. Selected genes in the cluster “Down” display high expression in oocytes and low expression in various other human tissues.

Two independent human preimplantation microarray datasets were analyzed in order to define genes with consistent gene expression profiles between different embryo stages [8], [11]. Only probes with significant changes in both datasets were included for further analysis, and classified into three clusters according to the expression pattern: “Up”, “Up-down” and “Down”. Probes in the cluster “Up” were upregulated between MII to 4-cell (958 probes in Zhang et al. 2009) or 1-cell to 4-cell (11 probes in Xie et al. 2010) or between 4-cell to 8-cell stages in both studies (454 probes in Zhang and 6112 probes in Xie). 336 probes corresponding to 295 different genes were significantly upregulated in both studies. Probes in the “Up-down” cluster were upregulated by 4- or 8-cell stages and were then downregulated by 8-cell or by the blastocyst stages including 176 probes common in both datasets (472 probes in Zhang, 1243 probes in Xie), corresponding to 156 genes. Probes belonging to the cluster “Down” were downregulated by 8-cell or blastocyst stages in both studies (8319 probes in Zhang, 7520 probes in Xie) including 2474 common probes corresponding to 2025 genes. A list of genes in all clusters is shown in Table S1, and examples of genes in each cluster are shown in Figure 1.

### Selection of genes for comparison between mouse and human

We selected genes from each cluster “Up”, ”Up-down” and “Down” for analyzing the expression profile of mouse preimplantation embryo by qPCR. Five different criteria for selecting these genes were applied (Table 1). First, the expression in various tissues was considered by using the Amazonia database [23] that combines microarray expression data from various human tissues and embryonic stem cells as well as from three different studies on human oocytes [5], [7], [24]. Genes with higher expression in oocytes compared to other tissues were preferentially chosen from the “Down” cluster (Figure 1). Second, we were interested in transcription factors that might play a role during early development. We used a combined list of transcription factors that was compiled from public databases [25]. Association with cancer was used as a third criterion in gene selection, because many early development related genes, such as NANOG, OCT4, SOX2, DPPA5A and STELLAR are relevant for cancer [26]–[29]. Fourth, we performed PubMed searches to find novel genes; a gene was considered novel if no publications were found for its function. A final inclusion criterion was expression in mouse preimplantation embryos. Mouse Genome Informatics (MGI) database contains cDNA source data for mouse early embryos [30], [31]. Mouse orthologs for the selected human genes were identified in the Ensembl database. A gene was included if its ortholog was found in any of the following samples in MGI: oocyte, unfertilized oocyte, fertilized oocyte, 2-cell embryo, 4-cell embryo, 8-cell embryo, 16-cell embryo, morula or blastocyst.

**Table 1:** Studied genes according to their selection criteria.

Criteria/Expression cluster	Up	Up-down	Down
**Expressed highly/specifically in oocytes (Amazonia)**			*NLRP11, NLRP4, NLRP7, NLRP9, TRIM62, RBM18, TAF5, SSXIP, TAF4, C17ORF79, BRPF1, IPO8, NLRP13, C21ORF7, ZBTB10, ZNF280C, ZNF618, ZHX1, FAM222B, IPO8, NLRP5, ZBTB49, NUDCD1, PRDM4, SFMBT1, TM2D3, KLHL20, C2ORF34, ZCCHC2, ZNF280B, ZSWIM3, PPP1R35, BRD1*
**Transcription factor**	*SFPQ, ZNF639,*	*PHF20, SSX2, SSX3, SSX4/SSX4B*	*TAF4, TAF5, C21ORF7, C21ORF59, BRPF1, ZBTB10, ZHX1, ZHX3, PRDM4, SFMBT1, BRD1*
**Associated with cancer**	*SFPQ, ZNF622, ZNF639, DDX39A, SRSF7*	*KLF17, KLHL11, SSX2, SSX3, SSX4/SSX4B, MAGEA2*	*SERPINB5, SSX2IP, ZBTB10, NUDCD1, ZNF280B, BRD1*
**Novel, no functional studies**	*C1ORF52*	*PRAMEF12, C21ORF91, PRAMEF1, PRAMEF10, PRAMEF4, KHDC1L*	*NLRP11, NLRP9, RBM18, TRIM61, TRIM62, C17ORF79, KLHL32, C21ORF59, NLRP13, ZNF280C, ZNF618, FAM222B, ZBTB49, TM2D3, C5ORF34, ZCCHC2, ZNF280B, ZSWIM3, PPP1R35*
**Expressed in mouse preimplantation embryo (riken)**	*SNRPA1, SFPQ, C1ORF52, CHTOP, ZNF622, ZNF639, CPSF6, DDX39A, SRSF7*	*MAGEA2, HIPK3, PNRC1, KHDC1, KLF17, KLHL11, PRAMEF12, PHF20, ZFYVE1, ZSCAN5A, TRIM43, PRAMEF1, PRAMEF10, PRAMEF4, KHDC1L*	*NLRP4, TRIM62, RBM18, NLRP9, TSPAN5, SERPINB5, SNRNP70, SSXIP, TAF4, TRIM61, BAZ1A, IPO8, C17ORF79, CLIP4, C21ORF59, ZBTB10, ZNF618, ZHX1, ZHX3, IPO8, NLRP5, PRDM4, CXORF40A, NUDCD1, PRDM4, TM2D3, SFMBT1, SUDS3, C5ORF34, KLHL20, ZCCHC2, ZNF280B, ZSWIM3, BRD1*

**Figure 2:**
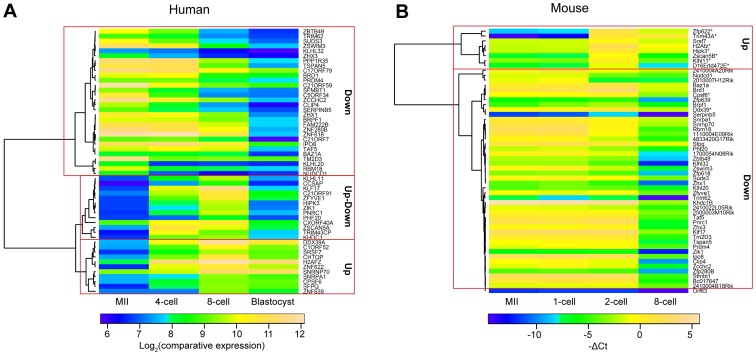
Clustering of selected human genes and their orthologs in mouse. Unsupervised clustering created three distinctive classes for human genes: “Up”, “Up-down” and “Down” (A). Mouse orthologs did not cluster similarly, but had a large cluster with mostly downregulated genes and a small cluster with upregulated (or up- and downregulated) genes (B). Average log_2_(comparative expression) values for each stage were used for the human data obtained from Zhang et al. (2007) microarray expression dataset and average −ΔCt values were used for the mouse expression data produced in the current study. Undetected samples were attributed the −ΔCt value of −14.8. Asterisks indicate mouse orthologs of human “Up” and “Up-down” cluster that were significantly upregulated in mouse between 1-cell and 2-cell stages.

All information was curated manually and 55 genes with orthologs in mouse were selected for gene expression profiling. The selection included 11 genes in “Up”, 14 in “Up-down” and 30 in “Down” cluster. Human microarray data from Zhang et al. (2009) were used for unsupervised clustering and plotting a heatmap (Figure 2A). In addition, members from the SSX, PRAMEF and NLRP gene families were selected for profiling in mouse. All the selected genes with their respective inclusion criteria are shown in Table 1. A list of the genes, the corresponding microarray probesets, mouse orthologs, mouse TaqMan assay names and expression values is in Table S1.

### Investigating gene expression during mouse early development

**Figure 3:**
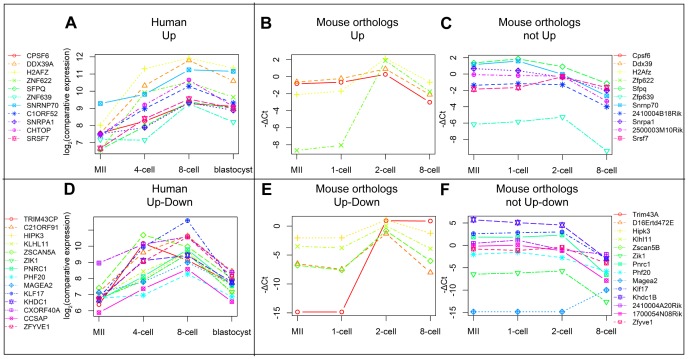
Comparison of early upregulated genes in human and mouse. Expression values for the genes in cluster “Up” for humans (A) and their orthologs in mouse show similar (B) and different (C) expression pattern between the two organisms. The “Up-down” cluster genes in human (D) and their mouse orthologs also show similar (E) and different (F) expression pattern. Similarly expressed orthologs were upregulated (p-value <0.05) from 1-cell to 2-cell stages (cluster “Up”) in mouse and downregulated from 2-cell to 8-cell stages (cluster “Up-down”) with the exception of *Trim 43a* which was only up-, but not downregulated. Maternal expression pattern was observed for differently behaving orthologs (C, F), which were downregulated by the 8-cell stage with the exception of Magea2. Average log2(comparative expression) values for each stage were used for the human data obtained from Zhang et al. (2007) microarray expression dataset and average −ΔCt values were used for the mouse expression data produced in the current study. Undetected samples were attributed the −ΔCt value of −14.8.

We studied the expression patterns of the selected genes in mouse. Custom TaqMan Low Density Array Cards (TLDA) were used for detecting the expression in the following mouse preimplantation stages: MII oocytes, 1-cell, 2-cell and 8-cell embryos. Five ng of RNA per sample was used in the first experiment in three biological replicas using TaqMan custom pre-amp pool for pre-amplification of cDNA with this approach. However, many assays did not pass our quality control criteria (Figure S1A). The experiment was then repeated with 12 ng of RNA per sample and no pre-amplification step. The quality of amplification curves was improved comparing to the pre-amplified samples (Figure S1B). 69 assays were analyzed in total, 4 were used as controls, and 1 assay was rejected for technical reasons. The upregulation control *Nanog* was detected only in the 8-cell stage as expected and the downregulation control *Nlrp5* decreased significantly from MII to 8-cell stage. *Psmb6* and *Hprt1* were used as endogeneous controls for normalization. Two (MII and 1-cell) or three (2-cell and 8-cell) biological replicas were used per developmental stage. Expression values were obtained using the comparative ΔCt method. A heatmap plotted for the selected genes is shown in Figure 2B. Mouse orthologs clustered remarkably different from the selected human genes (Figure 2). Human genes clustered into three groups based on the previous analysis, but mouse genes clustered as one downregulated group containing most genes, and a smaller group for genes that were significantly upregulated between 1- and 2-cell stages. Twenty-nine out of 30 orthologs for the genes in cluster “Down” were downregulated in the course of preimplantation development (p-val<0.05) similar to human, but only nine genes out of 25 in clusters “Up” or “Up-down” were upregulated by 2-cell stage in mouse. The human genes and mouse orthologs for “Up” and “Up-down” clusters are shown in Figure 3. Four and five orthologs in the human clusters “Up” and “Up-down”, respectively, were upregulated by the mouse 2-cell stage that is similar to human ZGA 4- to 8-cell stages (Figure 3B, E). However, seven and nine orthologs in the respective clusters were not upregulated by the 2-cell stage, but only downregulated by the 8-cell, except for *Magea2* and *2410004A20Rik*. Overall, more than half of the mouse orthologs for genes in “Up” and “Up-down” clusters shared the maternal gene expression profile being present already in the oocyte and downregulated later.

The methods for the human microarray experiments included poly(T) priming for cDNA [8], [11], whereas random hexamers were used for cDNA synthesis in the current study.

To exclude a possible bias caused by differences in cDNA priming, the experiment was repeated with 5 ng RNA in each stage in triplicate using poly(T) primers. The data were overall consistent, with Pearson correlation coefficients between 0,883 and 0.967 for comparisons of average −ΔCt values between the same stages (Figure S2). The p-values for genes in “Up” and “Up-down” clusters were not significant between 1-cell and 2-cell stages for *Cpsf6*, *Ddx39*, and *Hipk3*, although the trend for upregulation persisted (Figure S3). All other differentially regulated genes still had the maternal expression pattern in mouse, supporting the concept of differentially regulated genes.

Another difference between human and mouse embryos is the culture conditions of the embryos. Culture medium has been shown to influence gene expression in mouse early embryos [32]. In order to further confirm our conclusions, a comparison was made using a recently published RNA sequencing dataset on human and mouse oocyte and blastomere cells and preimplantation embryos [10]. The human embryos in the Xue et al. (2013) study were frozen, thawn, fertilized and cultured by using different protocols compared to Zhang et al. (2009), and the mouse eggs and embryos were obtained differently from the current study. The RPKM values of the Xue dataset were analyzed as described in Materials and methods. The selected genes in “Up”, “Up-down” and “Down” categories were extracted from both mouse and human sequencing datasets. A comparison of human and mouse genes between different methods is shown in Figure S4. The lowest correlation was observed for the 4-cell stage in humans (R = 0.366) and for the 2-cell stage in mouse (R = 0.543). This might result from the rapidly changing global gene expression patterns in these stages, requiring exact timing for embryo collection for better correlation between different studies. The *SNRPA1, SFPQ AND ZNF639* genes in the “Up” cluster were not significantly upregulated by 4- or 8-cell stage in this dataset, although the trend remained (Figure S5A). Surprisingly, only C21ORF91, HIPK3, ZIK1 and KLF17 belonged to the “Up-down” cluster in both datasets, while Trim43, KLHL11, ZSCAN5A, PNRC1, PHF20, KHDC1, CXORF40B and CCSAP showed no significant expression changes in the sequencing dataset (Figure S5D). A further look on the data showed that although the changes in expression were not statistically significant, all of the genes in the “Up-down” cluster still shared the same trends of upregulation by 4- or 8-cell stage and downregulation by the morula stage. All genes that were similarly upregulated in the mouse in TaqMan array dataset were also upregulated between the 1-cell pronuclear and 2-cell stages (Figure S5B, E). Differences occurred in genes that had maternal expression in TaqMan array, but were upregulated in the mouse sequencing dataset: *Snrpa1*, *2500003M10Rik, Sfrs7* and *Zfp639* in the “Up” and *Khdc1b, Zfyve* and *Pnrc1* in the “Up-down” cluster.

To expand on the described differences, we decided to analyze more highly upregulated genes in humans and their orthologs in mouse in the sequencing dataset. Only genes with more than 5 times overexpression by 4- or 8-cell stages in human compared to the oocytes (p-val<0.05) were used, resulting in 412 and 1010 upregulated genes, respectively. The orthologs in mouse were identified using the Biomart database, resulting in 324 and 857 genes, respectively. Heatmaps for the upregulated genes in human 4-cell and 8-cell stages are shown in Figures S6A and S7A). Both gene sets containing mouse orthologs clustered into two: upregulated and not upregulated (Figures S6B and S7B). This expanded analysis suggested that even more differences in early upregulated genes between human and mouse exist.

**Figure 4:**
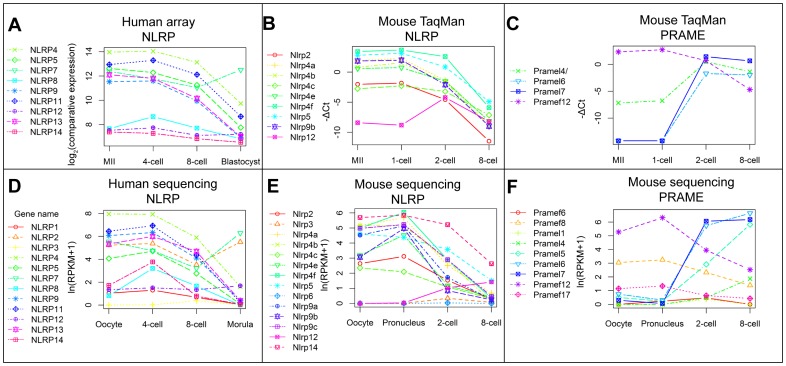
Expression profiles of NLRP and PRAME family genes in human and mouse. Most NLRP family members in the human microarray data share maternal expression pattern or are expressed at low levels (A). Mouse NLRP family orthologs in the TaqMan array share the similar maternal expression pattern, except for Nlrp12 (B). One gene in the mouse PRAME family is maternally expressed, Pramef12, while others are upregulated after fertilization (C), consistenly with the human PRAME family members on Figure 5. The results are supported by sequencing data by Xue, et al (2013) for human and mouse NLRP families (D, E) and mouse PRAME family (F). NLRP7 is upregulated after human 8-cell stage in both the microarray and sequencing dataset, in contrast to overall trend in the family (A, D). Mouse Nlrp12 is lowly expressed in both mouse datasets while the other genes are mostly higher expressed in the oocyte and 1-cell embryo (B, E). Mouse Pramef12 gene is maternally loaded in both TaqMan array and sequencing method in contrast to the rest of the genes in the family (C, F). Average log_2_(comparative expression) values for each stage were used for the human data obtained from Zhang et al., 2007 microarray expression dataset and average −ΔCt values were used for the mouse expression data produced in the current study. Undetected samples were attributed the −ΔCt value of −14.2. Human and mouse sequencing data from Xue, et al, 2013 shows average ln(RPKM+1) values for the same biological stage.

Genes belonging to developmentally interesting gene families were analyzed separately. The NLRP family members in human array and sequencing dataset were mostly downregulated, with the exception of NLRP7, which was upregulated after 8-cell stage in both datasets (Figure 4A, D). The NLRP family members in mouse were also downregulated in the course of time with the exception of Nlrp12 that was low expressed overall (Figure 4B, E).

**Figure 5:**
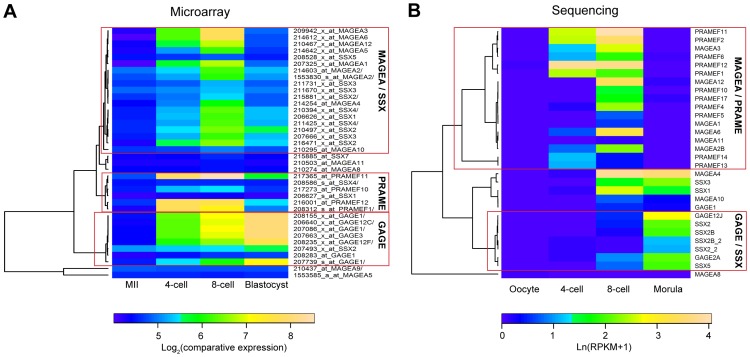
Expression of cancer-testis antigen family members in human preimplantation development. Human microarray data shows clustering of MAGEA/SSX and PRAME families into Up-down expression pattern (A). Sequencing dataset shows overall similar expression pattern for these families (B). GAGE family members are upregulated at later stages in the preimplantation embryo in both datasets. The microarray results are displayed as average log_2_(comparative expression) and the sequencing data as average ln(RPKM+1) for similar biological stages.

All the available *PRAME* and many SSX, MAGEA and GAGE family members in the human microarray belonged to the “Up-down” cluster and were highly upregulated between MII to 4-cell or 8-cell stages (Figure 5A). Similar “Up-down” expression pattern was observed for these gene families in the human sequencing dataset (Figure 5B), where the MAGEA and PRAME family genes clustered separately into “Up-down”. Most MAGEA and SSX family genes shared a common cluster for “Up-down” genes in the microarray dataset, but a separate clustering was seen for GAGE (microarray) or GAGE and SSX (sequencing) family genes that were upregulated also in the later stages (blastocyst and morula). The two datasets included many but not necessarily exactly the same members from both families. However, genes in all the selected families had dynamic expression profiles in the preimplantation human embryo.

The PRAME and SSX family genes were assessed in the mouse. Unfortunately, all assays failed to detect product in SSX family genes and there was no annotation of SSX family genes in the sequencing dataset. Three out of four PRAME family members in the mouse were upregulated by the 2-cell stage and Pramef12 had a maternal expression pattern (Figure 4C, F). The mouse Pramel6 and Pramel7 genes were most highly upregulated in both TaqMan array and sequencing datasets (Figure 4C, F). Pramel5 and Pramel4 were also upregulated, but they were not distinguishable in the TaqMan array dataset.

## Discussion

### Similar gene expression profiles between mouse and human

We identified selected genes relevant for human preimplantation development and studied orthologous gene expression in the mouse. We used two independent microarray datasets to identify differentially regulated genes in human preimplantation development. Five criteria were applied and 69 selected genes were successfully assayed for expression profiling in the mouse. Many of these genes had similar expression patterns between mouse and human in the course of preimplantation development; we found no changes in gene expression between MII oocyte and 1-cell stage in mouse nor between MII and zygote in humans.

Most genes in the NLR family, pyrin domain containing (*NLRP* family), were downregulated both in human and mouse. Most *NLRP* family genes, including *NLRP5* (*Mater*) are maternally loaded in human oocytes and downregulated by blastocyst stage [33]. Our results show that most mouse genes in the *NLRP* family were downregulated similar to human (Figure 4. B, E). We saw no differences in expression between mouse and human for 29 other genes in the “Down” cluster (Figure 2.A, B). This similarity of expression between human and mouse genes in the cluster “Down” was further supported by a comparative microarray study that showed consistent expression patterns between human and mouse for almost 70% maternally deposited transcripts, whereas only 40% of transcripts upregulated by ZGA displayed a similar expression pattern [11].

### Differentially regulated genes between human and mouse

We studied 25 genes that were upregulated in human by the ZGA at 4- and 8-cell stages. Nine of those were similarly upregulated, but sixteen were not. We found 7 and 9 genes from the classes “Up” and “Down”, respectively, that were not upregulated in the mouse ZGA stage in 2-cell embryos. All these genes, except for *MAGEA2*, were downregulated between 2- and 8-cell stages, showing a maternally loaded expression pattern. These include the transcription factors *SFPQ*, *ZNF639* and *PHF20* (Figure 2, Table 1). This difference did not depend on polyadenylation (Figure S3) nor on cell culture and analysis methods (Figure S6 and S7).

The three transcription factors *SFPQ*, *ZNF639* (also known as *ZASC1*) and *PHF20* have been associated with cancer [34]–[41]. *SFPQ* is an essential pre-mRNA splicing factor required early in the spliceosome formation [42]. Two other splicing factors in our study, *SNRNP70* and *SNRPA1* were upregulated in humans by ZGA, but maternal and downregulated in mouse. Zygotic transcription in mouse starts one day earlier than in human, perhaps suggesting earlier requirement for the splicing factors in the mouse.

A microarray study by He et al. (2010) suggested global differences in the mouse and human early gene expression, while a sequencing study by Xue et al. (2013) proposed similar expression. However, Xue et al. used stage-specific modules as the basis of their analysis, thus looking at gene expression values at different time-points as opposed to expression changes between stages. He et al. analyzed gene expression changes between stages and compared gene ontology categories. Neither of the studies compared expression profiles of differentially expressed genes between mouse and human. Our approach permitted the detection of specific gene clusters with differential expression profiles between mouse and human that have not been described before. In addition, we verified the observed patterns in the dataset from Xue et al. (Figure S6 and S7).

Differences in gene expression between the human and mouse preimplantation development might in part account for the timing differences between these organisms. Mouse preimplantation development is faster than human, requiring 84–96 h to reach blastocyst stage while it takes 24–30 h more for human [43], [44]. Furthermore, ZGA starts at 1- to 2-cell stage in mouse and at 4- to 8-cell stage in humans. This might be due to the presence of necessary transcripts already in the oocyte stage in mouse, while the genes are not yet expressed in human. Three such maternal genes in mouse described in this study are involved in splicing, which might contribute to the difference in timing for development. The developmentally important lineage-specific marker proteins are detected at different stages in human and mouse embryos [45].

### Cancer-testis antigens expression in the human and mouse preimplantation

Cancer/testis (CT) antigens are a category of tumor antigens with mostly unknown functions that are expressed in various types of cancer but have their expression otherwise restricted to male germ cells in the testis [46], [47]. We investigated four CT antigen families with dynamic expression profiles in human: Preferentially Expressed Antigen in Melanoma (*PRAME*), Synovial Sarcoma X breakpoint (*SSX*), Melanoma antigen family A (MAGEA) and G-antigen (GAGE). *PRAME* is a CT antigen with unknown biological function [48]. Many human *PRAME* family genes are clustered in the genome [49] and *PRAME* family genes on the microarray (*PRAMEF1*/2, *PRAMEF10*, *PRAMEF11*, *PRAMEF12*) belonged to the “Up-down” gene cluster. Four genes of this family were investigated in the mouse: *Gm13102*, *Pramef12*, *Pramel6* and *Pramel7*. *Gm13102* is situated next to two more *PRAME* family genes in mouse called *Oog2* and *Oog3*. In the course of this study, 4 members of the *PRAME* family called *Oog1* - *Oog4* were shown to be expressed in early mouse embryos or oocytes [50], [51]. We found that 3 members of the family - *Gm13102*, *Pramel6* and *Pramel7* - were upregulated by mouse 2-cell stage and thus had similar expression pattern as their human counterparts. The remaining gene, *Pramef12* was not upregulated, but already present in mouse oocytes. The *PRAME* gene family was predicted to have a role in spermatogenesis due to the expression levels and positive selection in mammals [52]. Our analysis on human microarray and mouse qPCR in early embryos showed that the *PRAME* family genes were highly upregulated in early embryos and suggested a role for this family in preimplantation development.

*SSX* genes are known to be expressed in normal testis and different types of cancer [53]. Our data show that several members of the *SSX* family had “Up-down” expression profiles in human preimplantation development (Figure 5). In contrast, the GAGE family genes persisted longer in the preimplantation embryo compared to the other CT antigens, until the blastocyst stage (Figure 5). Consistently, GAGE and MAGE family members had been found as highly expressed in the trophectoderm of mouse preimplantation embryo [54]. Both GAGE and MAGEA family members were detected in the postimplantation human embryo, suggesting an important role for CT antigens in cell differentiation processes [55]. MAGEA family proteins were also detected in placentas, whereas GAGE family members were not [56]. We conclude that there is strong evidence for important role of CT antigens both in the pre- and postimplantation embryo.

## Conclusion

We selected 70 differentially regulated genes with possible importance in human preimplantation development and investigated their expression in the mouse oocyte, 1-cell, 2-cell and 8-cell embryos. We found small differences in the maternally expressed and downregulated genes between human and mouse. In contrast, we found a set of genes that were upregulated in humans but not in mouse after zygotic genome activation. Sixteen out of 25 the genes in human “Up-down” and “Up” clusters had this difference in expression. Fifteen mouse orthologs shared the expression profile with maternally expressed genes and were downregulated in the course of preimplantation development, but were upregulated in humans. This difference in gene expression between human and mouse early embryos might account for part of the different preimplantation time in humans compared to mouse or for the differences in splicing. In addition, we described high expression levels for four cancer-testis antigen family members in ZGA and later stages of human preimplantation development. We suggest that the CT antigens have a function in the early embryos. Our findings show significant differences in the expression between mouse and human, limiting the generalizations from mouse to human preimplantation development. Knowledge about model systems limitations is crucial when investigating a complex process such as human preimplantation development.

## Supporting Information

Figure S1 **Examples of inconsistent amplification curves in the pre-amplified samples.** Amplification curves for MII oocyte, 1-cell, 2-cell and 8-cell embryos in two or three replicas on pre-amplified (A) and not pre-amplified (B) datasets are shown. Pre-amplified assays were excluded from the analysis due to inconsistent amplification profiles (A).
(PDF)

Figure S2 **Correlation plots between Mouse TaqMan array with oligo(dT) and random hexamer priming.** cDNA synthesis from mouse embryos was performed by using two protocols: random hexamer priming and oligo(dT) priming. The average values for each assay in a specific stage was plotted against similar sample in the different protocol. The Pearsson correlation coefficients for the comparisons are plotted in each figure.
(TIF)

Figure S3 **Gene expression profiles of “Up” and “Up-down” genes in mouse orthologs by using oligo(dT) priming for cDNA synthesis.** Expression profiles are shown for orthologs of human “Up” and “Up-down” genes in the mouse by using oligo(dT). primers for cDNA synthesis. The orthologs are plotted according to their distribution in the Figure 3: similar to human (A, C) and not similar to human (B, D). Genes marked by an asterisk do not share the same statistical significance as the ones primed with random hexamers (Figure 3), however the trends for up- and downregulation remain unchanged. Average −ΔCt values are plotted for each stage using the TaqMan array dataset generated in this study when using oligo(dT) primers for cDNA synthesis. Undetected samples were attributed a −ΔCt value of −13.1.
(TIF)

Figure S4 **Correlation plots between human microarray and sequencing data, and between mouse TaqMan array and sequencing data.** Different datasets for human preimplantation genes correlate with each other for mouse TaqMan array and sequencing study (A) and for human microarray expression and sequencing study (B). Mouse −ΔCt values from TaqMan array data in the current study were correlated with the log_2_(RPKM+1) values from sequencing data from Xue, et al, (2012) (A). Human microarray log_2_(comparative expression) data from Zhang, et al. (2009) was correlated with ln(RPKM+1) sequencing study by Xue, et al. (2012) (B). Correlation plots were done for similar biological stages in both organisms.
(TIF)

Figure S5 **Gene expression profiles of clusters “Up” and “Up-down” genes in human, and their orthologs in mouse using the sequencing data from Xue et al. (2012).** Selected genes from the human clusters “Up” and “Up-down” and their orthologs are plotted according to their distribution on Figure 3. Cluster “Up” genes for human (A) and their mouse similar (B) or different (C) orthologs are plotted by using ln(RPKM+1) values from the human and mouse sequencing data. “Up-down” genes are plotted for human (D) and their mouse orthologs (E, F). Human genes that are not significantly upregulated by 4- or 8-cell in the human sequencing dataset are indicated by an asterisk. Mouse genes that are significantly upregulated in the current sequencing dataset, but not on the TaqMan array in Figure 3, are indicated by an asterisk.
(TIF)

Figure S6 **Expression profiles for upregulated genes by 4-cell stage in human and their mouse orthologs.** All genes that were at least 5 times upregulated in human sequencing data by the 4-cell stage (p-value<0.05), were used for expression profiling (A). Their orthologs in mouse clustered into two large expression clusters “Up” and “Down” (B).
(TIF)

Figure S7 **Expression profiles for upregulated genes by 8-cell stage in human and their mouse orthologs.** All genes that were at least 5 times upregulated in human sequencing data by the 8-cell stage (p-value<0.05), were used for expression profiling (A). Their orthologs in mouse clustered into one large expression cluster “Up” and a smaller cluster with mostly downregulated genes.
(TIF)

Table S1 List of all selected genes in humans, corresponding probesets from microarray, mouse orthologs, TaqMan Low Density Array assay names, ΔCt for mouse qPCR data (current study) and average comparative expression values as log_2_(comparative expression) for human microarray data from Zhang et al. (2009). Gene clusters: Affy ID-s, corresponding gene names, significant fold-changes between consecutive changes in the Zhang et al. and Xie et al., distribution into the clusters. TLDA analysis: TaqMan assay names, corresponding ΔCt values for all the replicas and stages for the 12 ng protocol primed with random hexamers. Human vs mouse comparison: Clusters, human gene names and their mouse homologs together with average expression values between replicas in all the different stages. Similarities and differences between the expression profiles are shown.
(XLS)

Table S2 List of upregulated genes in human and their mouse orthologs from Xue, et al. (2013). RPKM+0.1 values are shown for all human genes that are significantly and more than 5 times overespressed either in the 4-cell or 8-cell stage compared to the Oocytes (p-val<0.05). Their homologues in mouse and their expression patterns are shown as RPKM+1.
(XLS)
