# Supplementary figures and images for: Differences in Gene Expression between Mouse and Human for Dynamically Regulated Genes in Early Embryo
Source: PLoS One. 2014 Aug 4;9(8):e102949. doi: 10.1371/journal.pone.0102949 (PMC4121084; doi:10.1371/journal.pone.0102949)

**A**

Pre-amplified

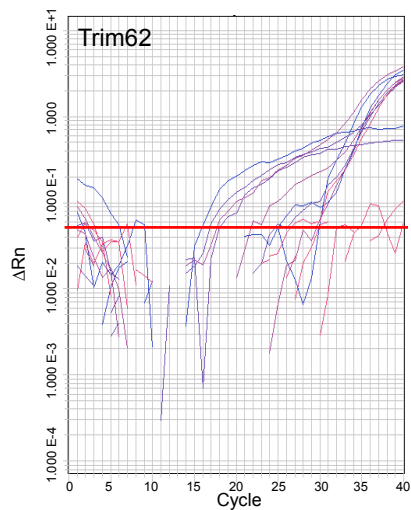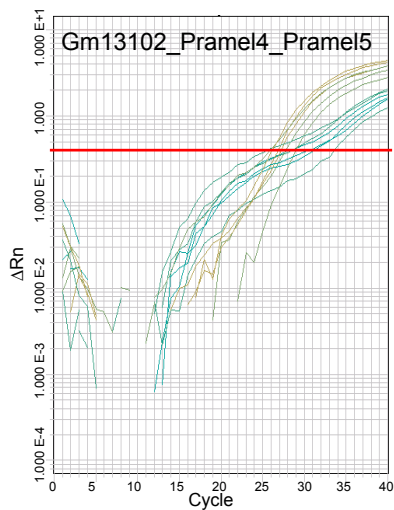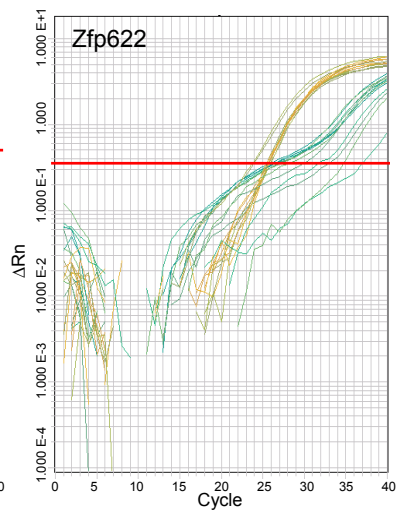**B**

Not pre-amplified

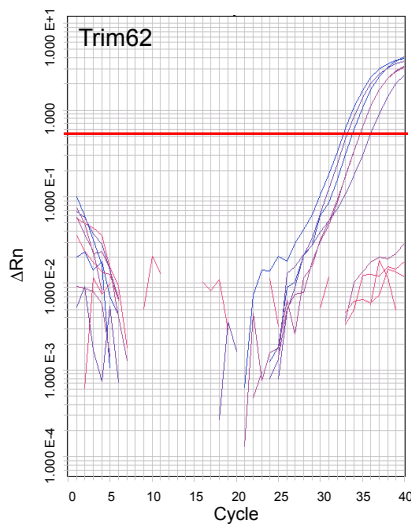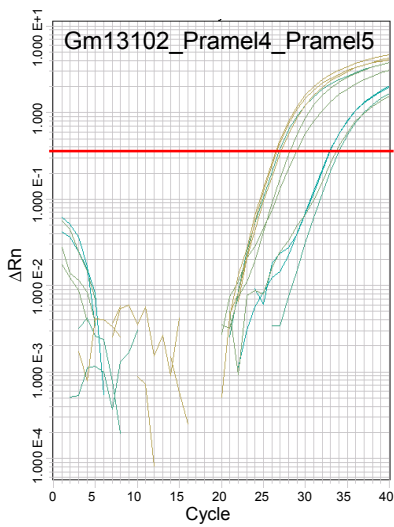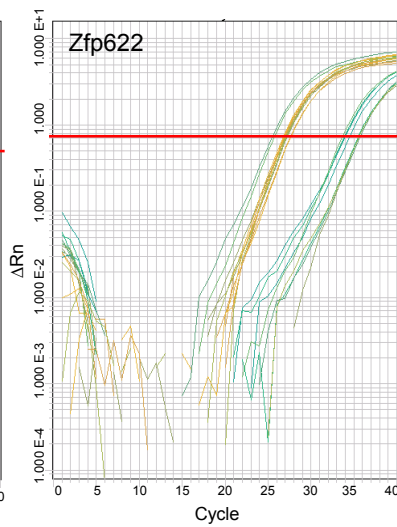

Supplement: Figure S1 — Examples of inconsistent amplification curves in the pre-amplified samples. Amplification curves for MII oocyte, 1-cell, 2-cell and 8-cell embryos in two or three replicas on pre-amplified (A) and not pre-amplified (B) datasets are shown. Pre-amplified assays were excluded from the analysis due to inconsistent amplification profiles (A). (PDF) [file pone.0102949.s001.pdf]

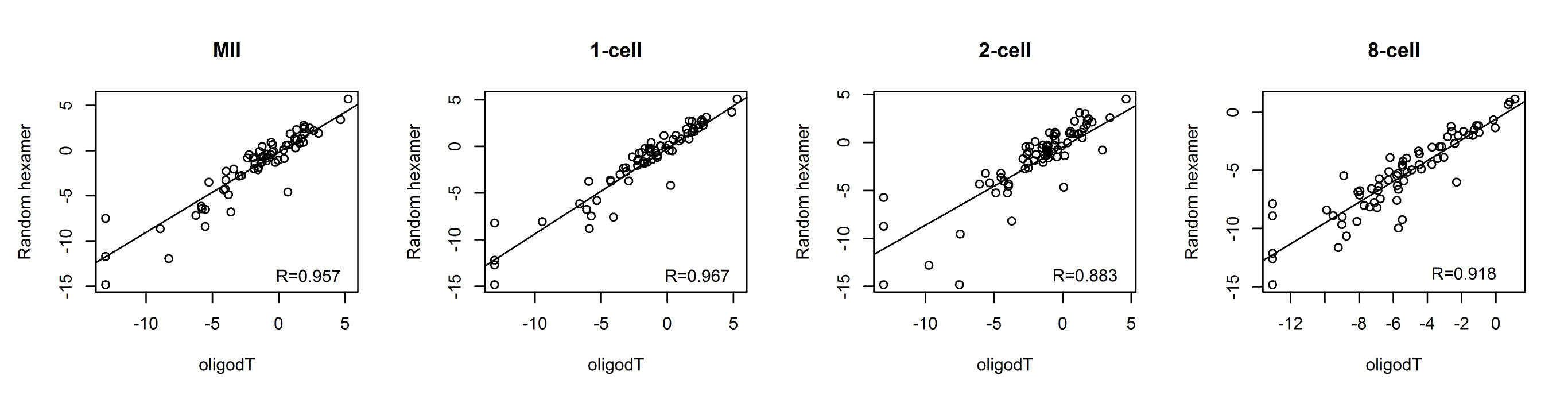

Supplement: Figure S2 — Correlation plots between Mouse TaqMan array with oligo(dT) and random hexamer priming. cDNA synthesis from mouse embryos was performed by using two protocols: random hexamer priming and oligo(dT) priming. The average values for each assay in a specific stage was plotted against similar sample in the different protocol. The Pearsson correlation coefficients for the comparisons are plotted in each figure. (TIF) [file pone.0102949.s002.tif]

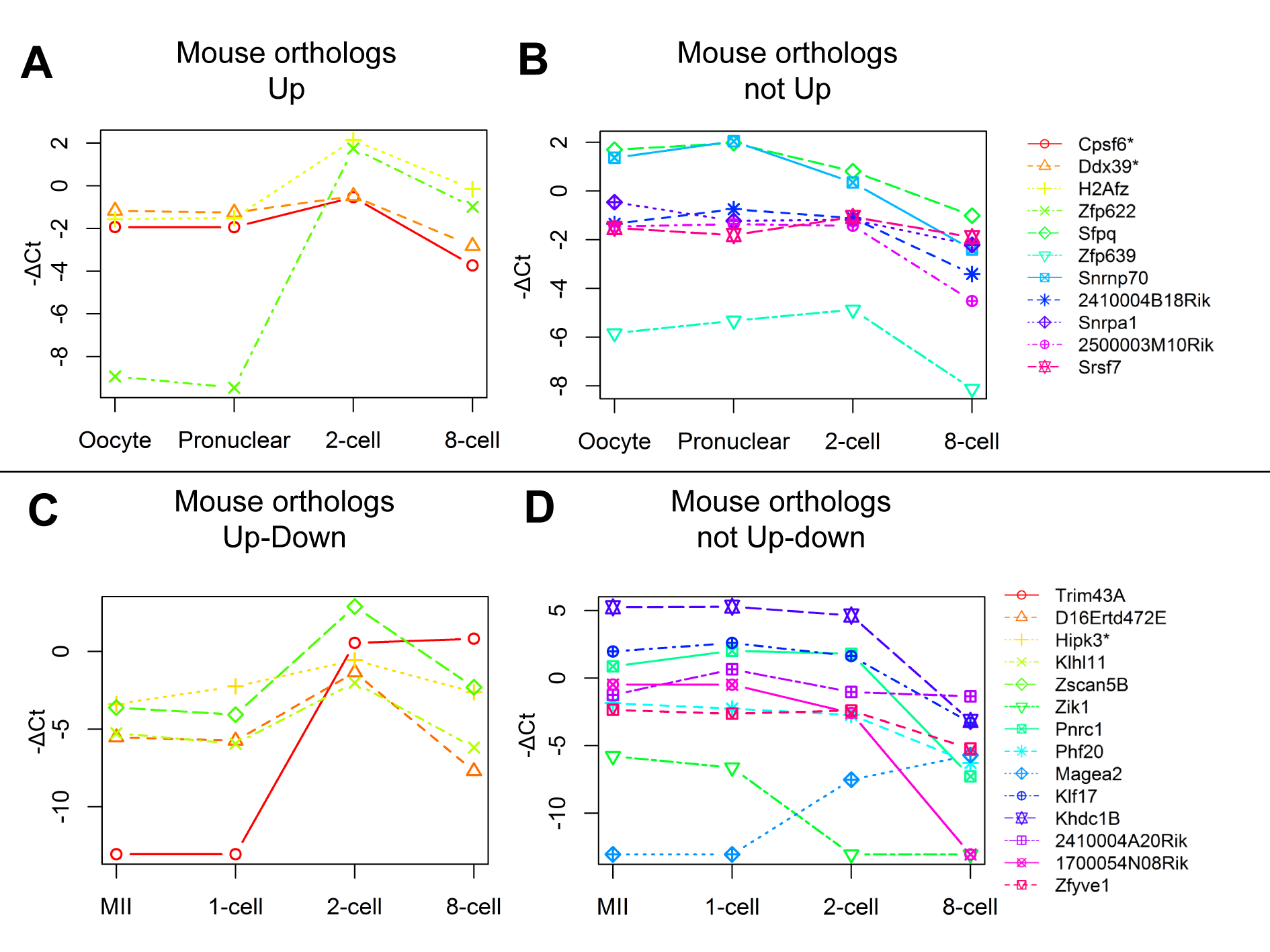

Supplement: Figure S3 — Gene expression profiles of “Up” and “Up-down” genes in mouse orthologs by using oligo(dT) priming for cDNA synthesis. Expression profiles are shown for orthologs of human “Up” and “Up-down” genes in the mouse by using oligo(dT). primers for cDNA synthesis. The orthologs are plotted according to their distribution in the Figure 3: similar to human (A, C) and not similar to human (B, D). Genes marked by an asterisk do not share the same statistical significance as the ones primed with random hexamers (Figure 3), however the trends for up- and downregulation remain unchanged. Average −ΔCt values are plotted for each stage using the TaqMan array dataset generated in this study when using oligo(dT) primers for cDNA synthesis. Undetected samples were attributed a −ΔCt value of −13.1. (TIF) [file pone.0102949.s003.tif]

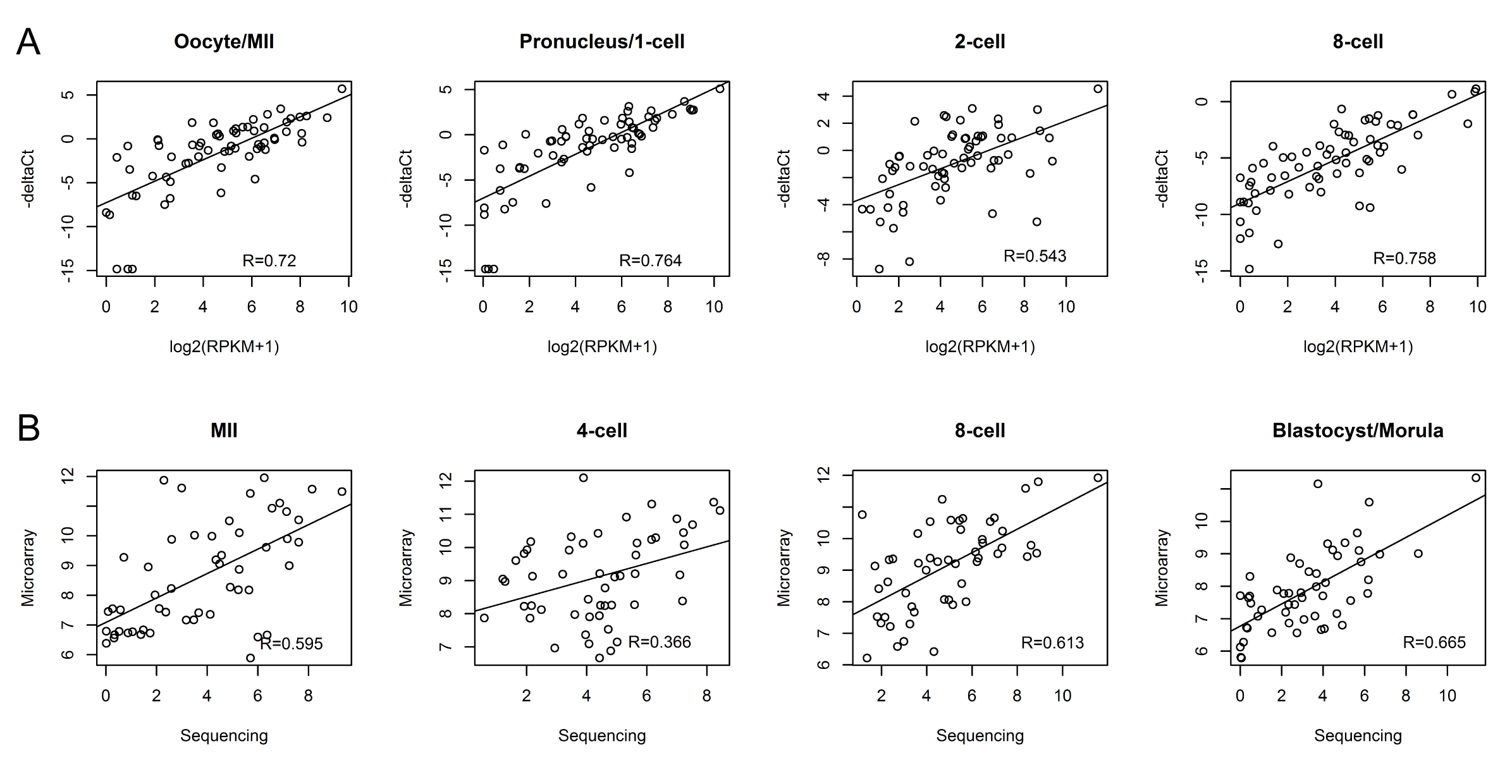

Supplement: Figure S4 — Correlation plots between human microarray and sequencing data, and between mouse TaqMan array and sequencing data. Different datasets for human preimplantation genes correlate with each other for mouse TaqMan array and sequencing study (A) and for human microarray expression and sequencing study (B). Mouse −ΔCt values from TaqMan array data in the current study were correlated with the log2(RPKM+1) values from sequencing data from Xue, et al, (2012) (A). Human microarray log2(comparative expression) data from Zhang, et al. (2009) was correlated with ln(RPKM+1) sequencing study by Xue, et al. (2012) (B). Correlation plots were done for similar biological stages in both organisms. (TIF) [file pone.0102949.s004.tif]

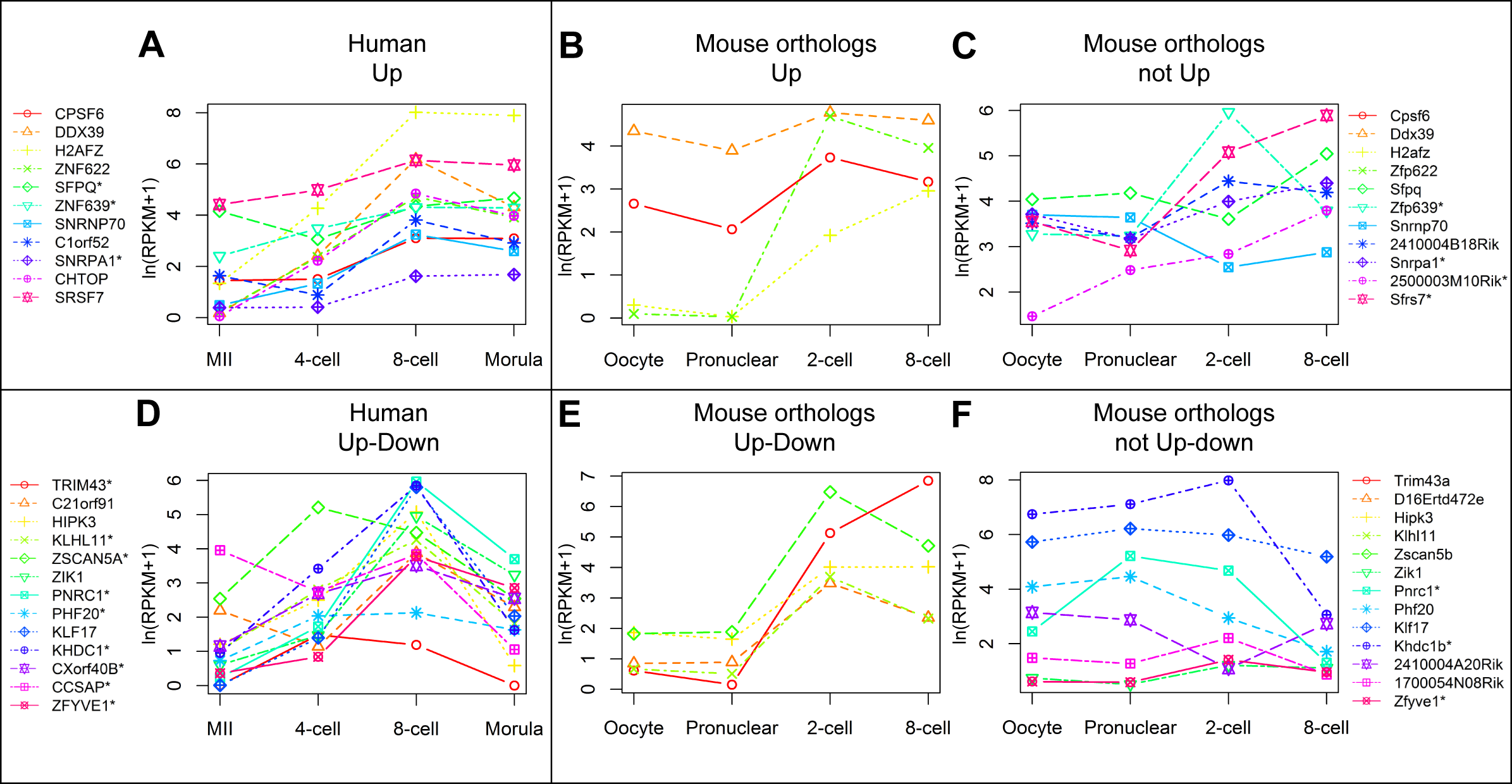

Supplement: Figure S5 — Gene expression profiles of clusters “Up” and “Up-down” genes in human, and their orthologs in mouse using the sequencing data from Xue et al. (2012). Selected genes from the human clusters “Up” and “Up-down” and their orthologs are plotted according to their distribution on Figure 3. Cluster “Up” genes for human (A) and their mouse similar (B) or different (C) orthologs are plotted by using ln(RPKM+1) values from the human and mouse sequencing data. “Up-down” genes are plotted for human (D) and their mouse orthologs (E, F). Human genes that are not significantly upregulated by 4- or 8-cell in the human sequencing dataset are indicated by an asterisk. Mouse genes that are significantly upregulated in the current sequencing dataset, but not on the TaqMan array in Figure 3, are indicated by an asterisk. (TIF) [file pone.0102949.s005.tif]

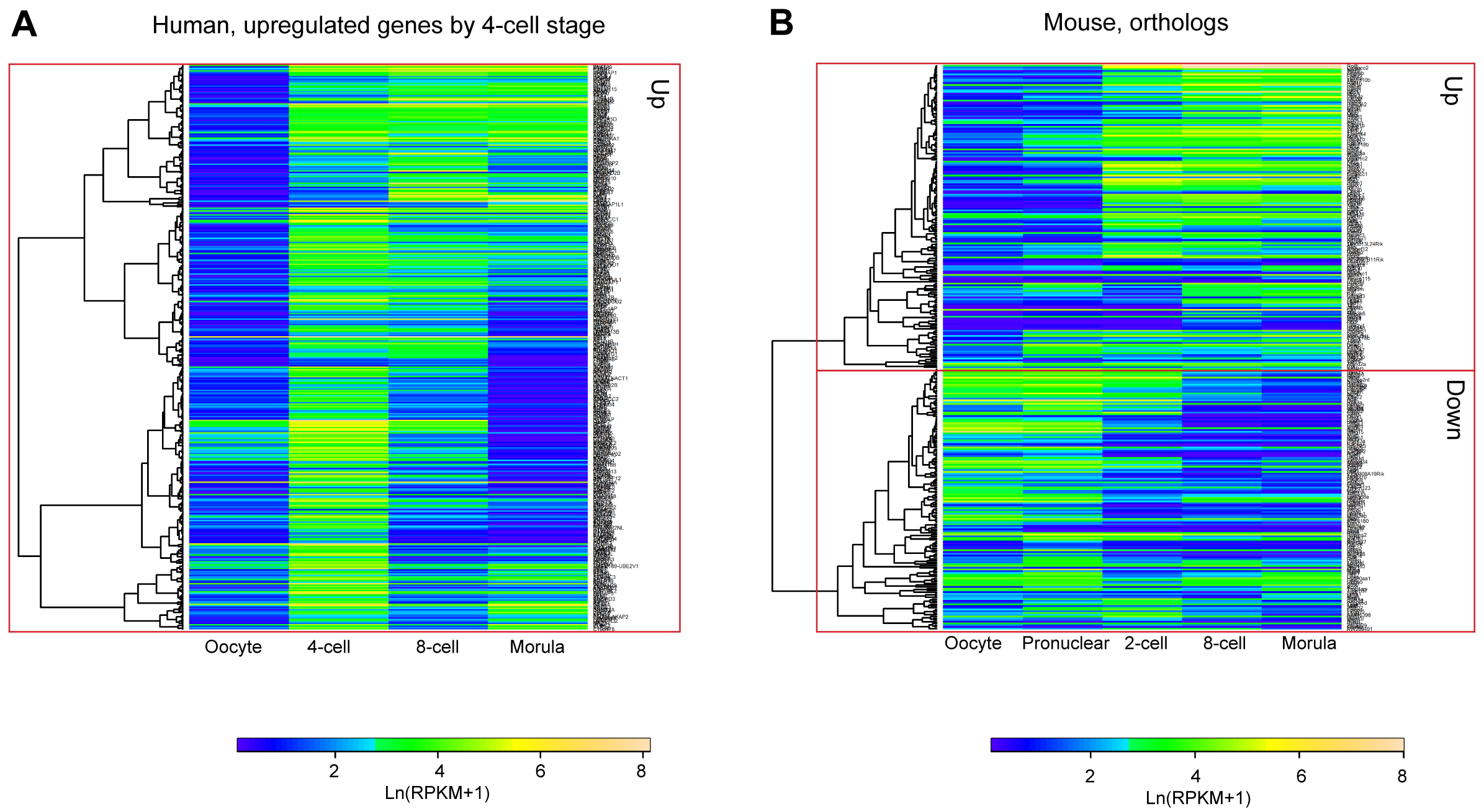

Supplement: Figure S6 — Expression profiles for upregulated genes by 4-cell stage in human and their mouse orthologs. All genes that were at least 5 times upregulated in human sequencing data by the 4-cell stage (p-value<0.05), were used for expression profiling (A). Their orthologs in mouse clustered into two large expression clusters “Up” and “Down” (B). (TIF) [file pone.0102949.s006.tif]

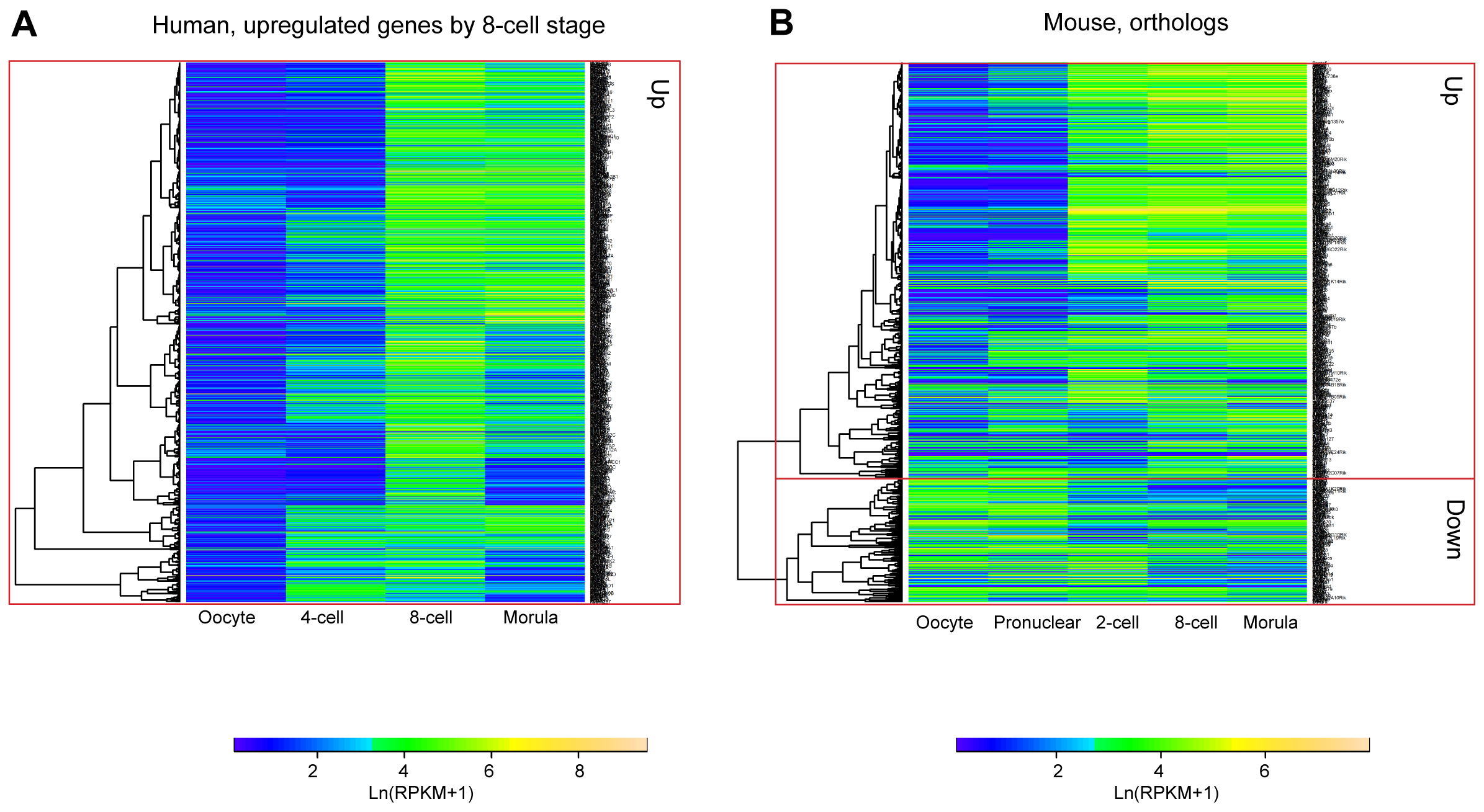

Supplement: Figure S7 — Expression profiles for upregulated genes by 8-cell stage in human and their mouse orthologs. All genes that were at least 5 times upregulated in human sequencing data by the 8-cell stage (p-value<0.05), were used for expression profiling (A). Their orthologs in mouse clustered into one large expression cluster “Up” and a smaller cluster with mostly downregulated genes. (TIF) [file pone.0102949.s007.tif]
